# Trust with Autonomous Intelligence Systems to Promote Adoption in Assistive Technologies: A Literature Review

**DOI:** 10.1093/geroni/igab046.2493

**Published:** 2021-12-17

**Authors:** Jared Carrillo, Maria Pena, Nonna Milyavskaya, Thomas Chan

**Affiliations:** 1 California State University Northridge, Van Nuys, California, United States; 2 California State University Northridge, Pacoima, California, United States; 3 California State University Northridge, Northridge, California, United States

## Abstract

While advancements in machine learning are increasing rapidly, very little progress has been made in its mass adoption despite its benefits in assistive technologies for older adults. By examining how users interact with smart technologies, characteristics of trust can be identified and enhanced to increase adoption of the next generation of assistive systems. The current study conducted a literature review to understand better how trust with autonomous systems is formed and maintained. Twenty-two pertinent articles were identified in which three themes emerged. First, people tend to forgive human errors more than errors made by machines -- meaning mistrust is exaggerated when systems make mistakes. Second, the development of trust depends on how the system solves the tasks it is assigned, for instance if a user does not believe the system acted in an “ethical way,” distrust may form and the continuation of adoption is decreased. Lastly, trust depends on the situation and the risk/reward associated with using the system, for example the trust needed to board an autonomous plane differs from that for a simple grammar correction. Taken together, the black box ideology of autonomous systems may be an issue that prevents trust in them to be formed and maintained. Promising future directions are to create machine language translators that improve transparency of autonomous system behaviors (i.e., explainability). Even if assistive technologies are created to aid older adults -- the lack of focus on understanding the factors that foster trust may dampen their actual use.

